# The Feasibility and Acceptability of LISTEN for Loneliness

**DOI:** 10.4236/ojn.2015.55045

**Published:** 2015-05-07

**Authors:** Laurie A. Theeke, Jennifer A. Mallow, Emily R. Barnes, Elliott Theeke

**Affiliations:** Department of Adult Health, School of Nursing, West Virginia University, Morgantown, USA

**Keywords:** Loneliness, LISTEN, Feasibility

## Abstract

**Purpose:**

The purpose of this paper is to present the initial feasibility and acceptability of LISTEN (Loneliness Intervention using Story Theory to Enhance Nursing-sensitive outcomes), a new intervention for loneliness. Loneliness is a significant stressor and known contributor to multiple chronic health conditions in varied populations. In addition, loneliness is reported as predictive of functional decline and mortality in large samples of older adults from multiple cultures. Currently, there are no standard therapies recommended as effective treatments for loneliness. The paucity of interventions has limited the ability of healthcare providers to translate what we know about the problem of loneliness to active planning of clinical care that results in diminished loneliness. LISTEN was developed using the process for complex intervention development suggested by the Medical Research Council (MRC) [1] [2].

**Methods:**

Feasibility and acceptability of LISTEN were evaluated as the first objective of a longitudinal randomized trial which was set in a university based family medicine center in a rural southeastern community in Appalachia. Twenty-seven older adults [(24 women and 3 men, mean age: 75 (SD 7.50)] who were lonely, community-dwelling, and experiencing chronic illness, participated. Feasibility was evaluated by tracking recruitment efforts, enrollment, attendance to intervention sessions, attrition, and with feedback evaluations from study personnel. Acceptability was assessed using quantitative and qualitative evaluation data from participants.

**Results:**

LISTEN was evaluated as feasible to deliver with no attrition and near perfect attendance. Participants ranked LISTEN as highly acceptable for diminishing loneliness with participants requesting a continuation of the program or development of additional sessions.

**Conclusions:**

LISTEN is feasible to deliver in a primary healthcare setting and has the potential to diminish loneliness which could result in improvement of the long-term negative known sequelae of loneliness such as hypertension, depression, functional decline, and mortality. Feedback from study participants is being used to inform future trials of LISTEN with consideration for developing additional sessions. Longitudinal randomized trials are needed in varied populations to assess long-term health and healthcare system benefits of diminishing loneliness, and to assess the potential scalability of LISTEN as a reimbursable treatment for loneliness.

## 1. Introduction

Diminishing loneliness has the potential to improve overall behavioral health by decreasing the risk for the development of depression [3] which could have major healthcare system benefits. Loneliness is a biopsychosocial stressor, associated with a physiological stress response [4] that is linked to multiple chronic conditions [5]-[7]. In addition, loneliness has been reported to be associated with suicidality [8] in younger populations and mortality in older populations [9]. The prevalence of loneliness is high with loneliness being experienced in up to 40% of older adults [10] and 16% of mid-life adults in the United States [11]. The descriptive literature on loneliness as a negative influence on human health led to a recent recommendation that loneliness be recognized a health determinant [12].

Since loneliness is rising as a health priority, it is critical that healthcare providers have access to effective interventions that could be employed in both community and clinical settings. Multiple reviews of interventions since the 1980s have concluded that no single intervention has demonstrated long-term effectiveness for diminishing loneliness [13]-[18]. The majority of intervention studies for loneliness have evaluated the effectiveness of interventions that were designed to target the social aspects of loneliness [19]-[23] and thus, emphasized enhancement of social skills, support, or integration. These interventions have included having participants engage in new activities as treatment for loneliness such as volunteerism [24] and friendship enrichment programs [22].

Interventions have been delivered to individuals, groups, and whole communities yet, to date, no intervention has demonstrated sustainability or scalability as an effective treatment for loneliness as a complex clinical health phenomenon. One meta-analysis of interventions for loneliness concluded that the most effective interventions were those that addressed the thinking errors that occurred when experiencing loneliness [25] such as automatic thinking of undesirability [26], perceptions of stigma [27], and persistent negative thoughts about self [28]. The findings from this meta-analysis were considered prior to the development of LISTEN. The purpose of this paper is to present the feasibility and acceptability of LISTEN (**L**oneliness **I**ntervention using **S**tory **T**heory to **E**nhance **N**ursing sensitive outcomes).

The Medical Research Council (MRC) framework for developing complex interventions [29]-[31] was used to guide the development of LISTEN [1] as the study team followed five sequential steps. The first step involved the intensive study of the health and social science literature on loneliness. This resulted in the decision that an intervention should target impaired thinking processes, be delivered in the group setting, and have the potential for both self-help and mutual group help with the possible benefit of befriending. The second step included evaluating multiple theoretical frameworks that would be supportive of this type of intervention. This theoretical review resulted in the decision that two different frameworks were necessary to build an adequate intervention for loneliness: story theory [32] and principles of cognitive restructuring [33] which are foundational to cognitive behavioral therapy. The third step included brainstorming with co-investigators and mentors to determine the most appropriate method to assess feasibility, acceptability, and effectiveness. It was determined that a randomized trial with an attention control group as an adequate comparator should be used. The fourth step was the first trial of LISTEN and the fifth step was the implementation of the evaluation plan to assess feasibility and acceptability of LISTEN.

LISTEN is a 5-session intervention that is delivered in 2-hour sessions over a sequential 5-week period with 1 session each week. The content for each session is guided by talking points that were determined from the literature on loneliness. The first session focuses on perceived belonging as the construct that matters most about loneliness to self. Unmet belonging has been reported as the antecedent of perceived loneliness [34] and belongingness support has been linked to diminished loneliness. The second session focuses on relationships, since perception of relationships is associated with loneliness in varied populations [35]-[37]. The third session focuses on role of one-self in the community by encouraging participants to discuss ways that they “get out” or “stay in”. This session is important because of the relationship between loneliness and functional decline. Inability to physically get out in community limits meaningful experiences and is linked to higher loneliness scores [38] [39]. Session 4 focuses on loneliness as a health challenge. During week 4, participants share ways that they meet the challenge of living with loneliness. During weeks 1 through 4, participants complete homework in preparation for the upcoming session. The fifth session is about establishing meaning in loneliness and identifying potential new solutions to loneliness as an individual health problem. During week 5, participants review progress made during weeks one through four and write messages for other people who might be experiencing loneliness.

## 2. Overview

This paper reports the feasibility and acceptability of implementing LISTEN in a sample of older adults. This study received a letter of approval from the West Virginia University Institutional Review Board. The longitudinal randomized trial was funded in September of 2011 by the Robert Wood Johnson Nurse Faculty Scholars Program and recruitment began in the Fall of 2011. Potential participants were recruited using flyers and posters that were placed in a family health primary care center. Volunteers called to seek participation and were screened over the phone for meeting inclusion criteria (age 65 years or older, UCLA Loneliness score > 40, community-dwelling, experiencing chronic illness). Once it was determined that inclusion criteria were met, an enrollment meeting was scheduled to complete informed consents and begin to gather baseline data. Participants received letters reminding them of their appointment to enroll. Once baseline data was collected, participants were randomized to either LISTEN group (N = 15) or attention control education group (N = 12) and scheduled to attend five weekly 2-hour sessions. Participants received reminder phone calls about the weekly sessions. At the end of the fifth session, participants completed anonymous evaluations of feasibility and acceptability. Participants were then scheduled for an appointment 12 weeks after the last session for final data collection and reminder phone calls regarding this appointment were made 1 week prior to the appointment. At the end of the final data collection, participants were given the opportunity to give additional verbal feedback to the study team regarding the implementation and evaluation of LISTEN as an intervention for loneliness.

### 2.1. Assessing Feasibility

The overall process of evaluating the feasibility of LISTEN was multifaceted. First, the study team tracked recruitment efforts, number of people who called seeking participation, screening processes for meeting inclusion criteria to participate, time to enrollment and participation, and completed enrollment. Second, once enrolled, attendance to intervention sessions was recorded so that “dose” of intervention could be evaluated. Attrition was tracked and plans were in place to track circumstances of leaving which included contacting participants after study completion to ascertain reasons for withdrawal. The study personnel were asked to provide written feedback about the process of delivering the intervention within a primary care setting, perceived strengths and weaknesses of delivering the intervention, and general comments about their perceptions of participant interest in the intervention as a sustainable and scalable method for diminishing loneliness.

### 2.2. Assessing Acceptability

Participants completed a quantitative and qualitative evaluation of the trial at the end of the fifth intervention session. Using a 5-item Likert scale, ranging from poor to excellent, participants ranked the usefulness, knowledge-building capacity, organization, clarity, study handouts, environment, hospitality of the study team, and overall experience. Participants were then asked to evaluate the length of each session, number of people in the groups, meeting times, and number of meetings as too long or too many, just right, or too short or too few. Participants were asked to identify and describe any burdens, barriers, or facilitators to participation. We also asked participants to give written feedback on the experience of being audio and video recorded during the study, any additional hindrances to participation, feelings about the opportunity to share feelings on loneliness, activities that were new since beginning the study, aspects of loneliness that they were not given the opportunity to discuss, key characteristics needed to lead the LISTEN intervention effectively, and ways to improve LISTEN. Field notes were kept by the study team for each intervention session and were used by the study team to further consider participant response to the intervention. Subsequently, at final data collection, participants were encouraged to give additional verbal feedback on their experiences during the trial.

## 3. Results

### 3.1. Feasibility

**Figure 1:**
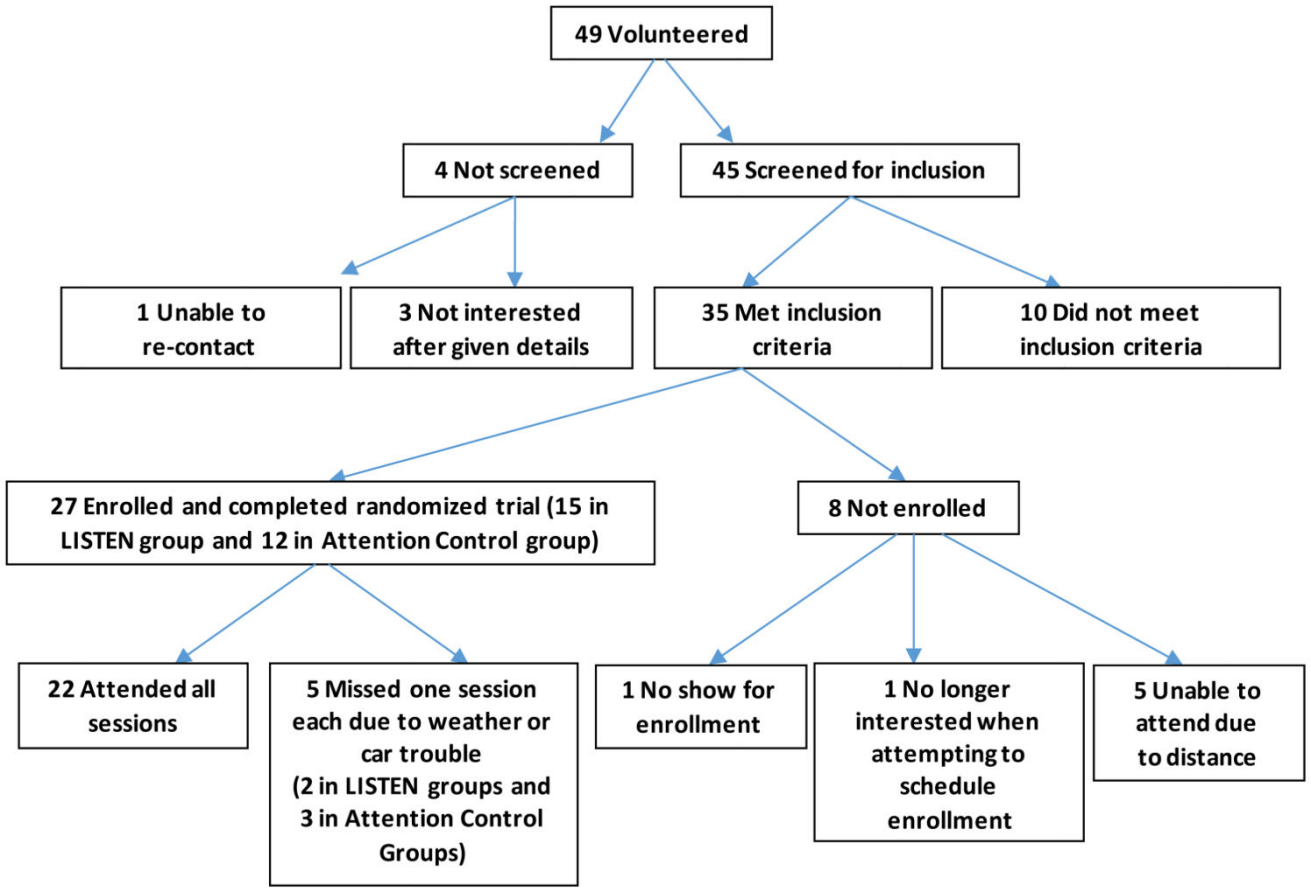
Summary of recruitment, enrollment, and completion by participants.

Figure 1 shows the flow of participants through the study. Recruitment to the study was feasible with 45 persons expressing an interest in participating during the first 3 months of recruitment. Enrollment was also feasible with thirty-five of these persons meeting the inclusion criteria and 27 enrolling in the study. Retention was not a problem and attrition was nonexistent. All 27 who initially enrolled completed the study. Absenteeism was low with only 5 participants missing one session each. Delivering the intervention was not difficult but did require significant preplanning including: parking accommodations that included an option of free valet parking, reserving rooms for the interventions sessions that facilitated audio/video recording, ensuring appropriate lighting for those with low vision, and providing seating for those with functional limitations (one participant required an elevated chair).

For this feasibility trial, the location was handicap accessible. The study team determined that delivering LISTEN to this specific population (older adults) could require a fully handicap-accessible location due to the possibility of decreased functional physical ability. Accommodations were made for those that could not write at the beginning of each session (which is part of LISTEN) by offering the option of recording to all participants. One participant did require the recorder but the other participants preferred to write while knowing they could record. Table 1 reports the sociodemographics of the participants.

### 3.2. Acceptability

Individuals who participated in both the LISTEN intervention groups and the attention control groups completed evaluations related to the acceptability. Overall evaluations were positive about the potential of LISTEN to be therapeutic for loneliness. Participants rated LISTEN as useful, contributing to new knowledge, clear, and organized. In addition, participants ranked the homework assignments for LISTEN as very useful. In general, participants rated the length of the sessions as “just right” with 2 participants rating them as “too short” and six of the 15 participants saying that 5 sessions was “too few”. Table 2 displays the evaluation scores from the LISTEN group participants.

After participants completed the quantitative component of the evaluation, we asked them to further evaluate by writing answers to additional questions. First, participants of LISTEN were asked to report any burdens, barriers, or hindrances to participation. Ten participants reported none. Three participants mentioned occasional difficulty with the weather, one participant mentioned distance to get to intervention, and one participant described that an episode of car trouble was a barrier. One participant wrote about a personal discomfort with feedback as a hindrance to actively participating in the group setting.

Second, when asked to identify factors that facilitated participation in the LISTEN groups, twelve participants identified at least one facilitator and three did not identify any. Six participants described a desire to express self as the most important facilitator. Three participants identified a desire to learn as influential to their participation. Two other participants reported that the openness of the group and the opportunity to talk to clear thoughts were important. Finally, participants wrote that the ease of parking and the escort to the study room were both facilitators.

Third, participants were asked to evaluate the experience of being audio/videotaped during the group sessions and all participants reported that the audio/videotaping was not intrusive or bothersome. One participant reported that she thought it would be useful for the study and she actually forgot the camera was running. Another said that the audio/videotaping was irrelevant to the group experience.

Fourth, participants of LISTEN groups were asked if they were fully able to express feelings of loneliness and they reported that they were “comfortable” and “felt accepted”. They reported that “it helped to get some things out in the open especially when you don't have anyone else to share those feelings with”. Participants wrote of the important role of others, describing that they “appreciated being” allowed to talk without interruption, “enjoyed others' views”, and “got good input from others”. Participants wrote about the group setting with statements including: “I was very happy to share my feelings”, “it felt good to open up to people in our generation and also to the young instructors”, “I felt that I was really being listened to”, and “I appreciated the fact that everything I shared was in confidence”. Finally, participants described how the group affected their thinking by writing: “it made me think more and not in a negative way as I usually do”.

Fifth, we asked the participants of LISTEN for feedback on the characteristics of an interventionist who might deliver LISTEN in the future and to tell us how we could improve LISTEN. When describing the necessary characteristics of future interventionists, participants used descriptive words about personality characteristics such as “warm, and accepting person”, “a good listener”, “empathetic”, “non-judgmental”, and “a person who respects all types of people”. They also emphasized the importance of intelligence about the topic and organizational skills by describing a person who “can keep people on track”, “has knowledge of relationship dynamics”, and “a good questioner”. When asked about improving LISTEN, participants commented that “writing first at the beginning of each session and then talking helped to organize my thoughts at the beginning”, “it was great the way it was”, “it doesn't need to be changed”, “we needed more time”, and “more sessions would still be helpful”.

Sixth, we asked participants if they had adopted new behaviors or activities that impacted their loneliness since the last session of LISTEN. Four participants had no new activities. However, the remaining participants were engaged in at least one new activity after learning about them from other members of the group. We received three reports of participating in a lifelong learning institute offered by the local university, three of increased volunteer service, two of joining new exercise classes, two of purposeful proactive planning to engage with family more often, one of more frequent church attendance, and one describing the beginning of a new creative artistic endeavor. Participants of the LISTEN groups did report an average decrease on the UCLA loneliness scale of 6 points when reassessed 12 weeks after the last intervention session.

Lastly, the attention control group (N = 12) received information in educational sessions about aging and these participants also identified weather, distance and car trouble as barriers to study involvement. Participants of this group identified that the leader needed to be knowledgeable and ten participants said that nothing needed to be changed. One participant suggested more sessions and one suggested that the first session was boring to them. One participant of this group identified that the participant gift card was an incentive for coming. We asked the participants to rank their learning for the five education sessions which included: Common Physical Changes with Aging, Eating for Health, Aging and Health, Stroke Prevention, and Preventive Care. For all of the sessions, participants ranked the session as successful for enhancing knowledge. The attention control group did not significantly diminish in loneliness but the overall experience of attending these educational sessions was rated positively by the participants. Table 3 displays the evaluation scores from the attention control education group.

## 4. Discussion

This study examined the feasibility and acceptability of LISTEN, a new intervention for loneliness, in a sample of chronically ill, community-dwelling, lonely older adults with moderate to high loneliness scores as measured by the UCLA Loneliness Scale [40]. Participants evaluated LISTEN as highly acceptable and commented that additional sessions could be useful. This feedback from participants will be used to refine LISTEN, potentially leading to the development of additional booster sessions or a second phase of LISTEN that involves sustained regular contact with participants. This could be accomplished in several ways including the possibility of using technology. The continued development of LISTEN will include this important adaptation for those who experience less loneliness but still are not recovered.

The three barriers to participation were travel distance, weather, and personal transportation issues. Five potential study participants were unable to enroll due to distance. This raises the awareness of location of intervention delivery, particularly in areas with underdeveloped major highway systems which make traveling even short distances more time consuming. Although few participants identified inclement weather and personal transporttation as barrier, in larger samples this could become problematic, particularly in areas with limited public transportation options. These identified barriers necessitate the future exploration of delivering LISTEN in community settings or via technology.

The importance of telling one's story was viewed as essential to the success of LISTEN by the participants. Having the opportunity to hear how others deal with the complicated health challenge of loneliness was described by participants as the most important part of participating. Participants reported a transformation in thinking based on the experience of telling and hearing stories of loneliness simultaneously. Hearing the vicarious experience of others as they coped with loneliness led to the adoption of new behaviors by study participants after completing participation in this study.

Feedback from participants will be used to consider the requisite characteristics needed to lead the LISTEN groups in larger trials. Characteristics like being accepting, organized, open to listening, empathetic, and respectful do not mandate that the interventionist be a formally trained health care professional. It may be possible that lay persons with these characteristics could be trained to understand the complex nature of loneliness and deliver LISTEN. It has been demonstrated that, with training, lay persons can deliver counseling that results in behavior change [41].

The rigorous process, the MRC framework, used in developing LISTEN [1] contributed to positive evaluations of feasibility and acceptability. The study team's familiarity with health and social science literature on loneliness was key to feasibility because the in-depth understanding of the findings in this literature led to conceptualization of LISTEN. In addition, the unique integration of two theoretical frameworks: the principles of cognitive restructuring [33] and concepts from story theory [32], provided the necessary components so that LISTEN could be acceptable. The use of Story theory in LISTEN is specific to the experience of loneliness. While Story theory has been used to develop interventions for other chronic illnesses such as hypertension [42] in each case, the intervention is designed by the interventionist to focus on the unique aspects of the specific health challenge. Most recent literature suggests that a combination of behavioral intervention and medication may be efficacious in the treatment of loneliness. LISTEN has the potential to be used as a unique therapy or concurrently with medications that have demonstrated effectiveness for loneliness in controlled trials [43].

## 5. Limitations

This study had several limitations. First, the study used a convenience sample of older adults that were well-educated and primarily female which may not truly be representative of the larger patient population of older adults. The higher numbers of females may have influenced the acceptability of the intervention because it has been reported that women may be more open to alternative therapies [44]. The study was conducted in a small city and several participants lived in rural counties so feasibility and acceptability may be different in samples of persons who reside in larger cities.

## 6. Conclusion

This study adds new knowledge about the feasibility and acceptability of LISTEN, a new intervention for loneliness. LISTEN has the potential to be a sustainable and scalable treatment for loneliness after future longitudinal trials are accomplished. The next step will be to conduct larger longitudinal trials of LISTEN to appropriately assess the long-term health and health system benefits of diminishing loneliness in varied populations. The study team has completed preliminary work on loneliness with stroke survivors [45], college students [7], children [46], and adults with multiple chronic conditions [47] [48], building a foundation of knowledge about loneliness for future studies of LISTEN.

## Figures and Tables

**Table 1:** Sample characteristics [N = 27 older adults, mean age 75 years (SD 7.5)].

Variable	Category	N (%)
Gender	Female	24 (89)
	Male	3 (11)
Marital status	Married, spouse in home	8 (30)
	Separated/divorced	10 (37)
	Widowed	8 (30)
	Never married	1 (3)
Education	High school diploma	7 (26)
	Some college	5 (19)
	College degree and higher	15 (55)
Household income ($/year)	$0 - $20,000	10 (37)
	$20,001 - $30,000	6 (22)
	$30,001 - $50,000	8 (30)
	$50,001 and up	3 (11)
Employment status	Retired	18 (67)
	Working part-time	6 (22)
	Working full-time	1 (3)
Number of chronic illnesses	One	6 (22)
	Two	6 (22)
	Three	9 (33)
	Four or more	6 (22)

**Table 2:** Evaluation scores for acceptability of LISTEN group participants (N = 15).

Concepts of LISTEN	Mean score (SD) [range 1 - 5]	Mode
Usefulness of LISTEN	4.8 (0.41)	5
New knowledge acquired	4.4 (0.74)	5
Organized	4.8 (0.56)	5
Clarity	4.8 (0.56)	5

**Environment of LISTEN**	**Mean score (SD) [range 1 - 5]**	**Mode**

Homework	4.5 (0.92)	5
Location	4.6 (0.83)	5
Hospitality	4.8 (0.56)	5
Overall	4.8 (0.41)	5

**Structure of LISTEN**	**Mean score (SD) [range 1 - 3]**	**Mode**

Session length	1.9 (0.35)	2
Time of sessions*	1.9 (0.35)	2
Number of sessions	1.7 (0.45)	2
Number in group	1.0 (0.0)	1

*Note*: Scales ranged from either 1 (poor) to 5 (very good) *or* 1 (too few or too early*) to 3 (too many or too late*).

**Table 3:** Evaluation scores for the attention control education groups (N = 12).

Concepts of education group	Mean score (SD) [range 1 - 5]	Mode
Usefulness of LISTEN	4.9 (0.29)	5
New knowledge acquired	4.6 (0.68)	5
Organized	4.9 (0.29)	5
Clarity	5.0 (0.00)	5

**Environment of control group**	**Mean score (SD) [range 1 - 5]**	**Mode**

Homework	4.9 (0.29)	5
Environment	4.6 (0.51)	5
Hospitality	4.8 (0.39)	5
Overall	5.0 (0.00)	5

**Structure of control group**	**Mean score (SD) [range 1 - 3]**	**Mode**

Session length	2.0 (0.00)	2
Time of sessions*	2.0 (0.00)	2
Number of sessions	2.0 (0.00)	2
Number in group	1.8 (0.39)	1

*Note*: Scales ranged from either 1 (Poor) to 5 (Very Good) *or* 1 (too few or too early*) to 3 (too many or too late*).
